# Disclosing the relationship between public service motivation and job satisfaction in the Chinese public sector: A moderated mediation model

**DOI:** 10.3389/fpsyg.2023.1073370

**Published:** 2023-02-16

**Authors:** Ying Zhang

**Affiliations:** School of International and Public Affairs, Shanghai Jiao Tong University, Shanghai, China

**Keywords:** public service motivation, job satisfaction, role overload, marital status, public employees

## Abstract

**Introduction:**

Despite the relationship between public service motivation and job satisfaction is widely discussed, rare studies explore the theoretical mechanism of this relationship.

**Methods:**

Through integrating public service motivation, role overload, job satisfaction, and marital status, this study explores psychological mechanisms and boundary conditions of the relationship between public service motivation and job satisfaction. Data was collected from 349 public employees in eastern China.

**Results:**

Empirical results reveal that public service motivation is positively related to job satisfaction by decreasing role overload. Moreover, marital status moderates the relationship between role overload and job satisfaction, as well as moderates the indirect effect of public service motivation on job satisfaction through role overload.

**Discussion:**

These findings advance our understanding of the psychological mechanism and conditional effect of PSM in relation to job satisfaction and provide valuable insights into how to improve public employees’ well-being.

## 1. Introduction

Since introduced by Perry and Wise (1990), public service motivation (PSM) has attracted growing attention from scholars and practitioners, which is considered an important antecedent of work attitudes and behaviors, such as job satisfaction (e.g., Liu et al., 2008; Homberg et al., 2015), organizational commitment (Crewson, 1997; Sun, 2021), performance (Bellé, 2013; Andersen et al., 2014), change-supportive behavior (Hassan et al., 2021), and organizational citizenship behavior (Liu and Perry, 2016; Chen et al., 2021). This study focuses on the relationship between PSM and job satisfaction. As the most frequently studied outcome of PSM, job satisfaction is of great interest. On the one hand, job satisfaction can predict employee well-being and is associated with health variables such as burnout, depression, and anxiety (Faragher et al., 2013; Di Marco et al., 2016). Also, from the humanitarian perspective, happiness or satisfaction is a permanent theme of life, and job satisfaction is a measure of “happiness at work,” which is worth pursuing in itself (Brown et al., 2012).

Nevertheless, the relationship between PSM and job satisfaction shows mixed or conflicting results. On the one hand, public employees who possess a high level of PSM are more likely to feel value consistency with their organization, perceive a high level of person-organization fit, devote themselves to public service and easily find job enjoyment, thus PSM will be positively linked to job satisfaction (Liu et al., 2008; Quratulain and Khan, 2015; Roh et al., 2016; Li et al., 2022). Meta-analysis of the relationship between PSM and job satisfaction also presents a positive relationship (Homberg et al., 2015). On the other hand, some scholars proposed that PSM might have dark sides, suggesting that employees with high PSM are prone to work overload, perceive more work stress, and suffer their well-being (Giauque et al., 2013; Liu et al., 2015a,b; Van Loon et al., 2015). Meanwhile, scholars have also found a non-direct or non-significant relationship between PSM and job satisfaction (Wright and Pandey, 2008, 2011; Rayner et al., 2018).

Moreover, previous research usually focuses on the direct relationship between PSM and job satisfaction. Only a handful of studies have explored the psychological process and conditional effect of these two constructs. For instance, scholars have studied the mediating effect of value congruence (Wright and Pandey, 2008), mission valence (Wright and Pandey, 2011), emotional labor strategy (Li and Wang, 2016), organizational commitment (Park, 2020), perception of organization prestige (Bright, 2021), as well as the moderating effect of love of money (Liu and Tang, 2011), person-organization fit and needs-supplies fit (Liu et al., 2015a,b). It can be seen that the extent of studies on the theoretical mechanism between PSM and job satisfaction is limited and mostly carried out from the perspective of person-organization fit.

To address the research gap, our study aims to replicate and retest the relationship between PSM and job satisfaction in the Chinese public sector and to expand previous literature by examining a moderated mediation model. Based on the Job Demands-Resources model, job characteristics can be divided into two categories: job demands and job resources. Their interaction plays a vital role in the development of job strain (Bakker and Demerouti, 2007). PSM belongs to personal resources, and public employees with high PSM can better cope with tremendous job demands (Giauque et al., 2013; Bakker, 2015). Role overload occurs when employees feel insufficient resources to fulfill assigned requirements, commitments, or obligations (Peterson et al., 1995). When faced with tremendous work demands, we argue that highly motivated public employees possess high person-organization fit, feel more support and resources, therefore, are less prone to role overload, and have a high level of job satisfaction. Meanwhile, Chinese public employees describe their work situation as “A thousand threads from above, one needlepoint below.” Role overload has been a prominent and imperative issue in the Chinese public sector and significantly influences employees’ well-being (Liu et al., 2015a,b). Hence, this study will empirically examine the mediating effect of role overload between PSM and job satisfaction. Family roles could interact with work roles. Marital status, as a valued life condition in the family role (Hobfoll, 1989), can provide resources for the work domain (Ten Brummelhuis and Bakker, 2012). Simultaneously, married employees might face increasing demand at home and require more energy and time (Vatharkar and Aggarwal-Gupta, 2020). Thus, marriage may affect employees’ perception of role overload and its influence on employees’ well-being (Byron, 2005). However, the impact of marriage may vary by national context. Traditional Chinese family values constitute the context of marital life (Hu and Scott, 2016). For example, the wife is thought to be the primary housework role due to traditional gender roles, and family members across different generations tend to live together because of filial piety (Shek, 2006). It is necessary to investigate the conditional effect of marital status in Chinese context.

Through examining the aforementioned relationships, this study has a twofold contribution. Firstly, this study has examined the mediating effect of role overload in the relationship between PSM and job satisfaction, advancing the knowledge of the psychological mechanism of PSM in relation to job satisfaction. Role stress, like role ambiguity and conflict, has received extensive attention, and less attention has been paid to role overload (Örtqvist and Wincent, 2006). To the best of our knowledge, it is the first study to empirically test the relationship between PSM and role overload, which enriches the literature on role overload. Secondly, this study explores the moderation effect of marital status and reveals that marriage plays a buffering effect. Through deepening understanding of resources in both work and family domains, this study contributes to the development of role stress theory and provides insights to improve employees’ job satisfaction.

## 2. Literature review and theoretical hypothesis

### 2.1. Public service motivation and job satisfaction

Job satisfaction is viewed as “a pleasurable or positive emotional state resulting from the appraisal of one’s job or job experiences” (Locke, 1976). It is believed that highly satisfied employees tend to possess higher organizational commitment (Aydogdu and Asikgil, 2011), have lower turnover rates (Zhang et al., 2022), perform more organizational citizenship behavior (Zeinabadi, 2010), as well as higher job performance (Judge et al., 2001). Nowadays, public sectors are faced with an increasingly volatile environment, and they have to improve employees’ productivity to meet the public service needs of citizens under the circumstance of personnel cutbacks and budget constraints (Piatak, 2019; Liu, 2020; Ahmad et al., 2021). Maintaining employees’ job satisfaction is one of the prerequisites for improving organizational effectiveness and providing high-quality public service (Cantarelli et al., 2016). However, public employees have low job satisfaction (Johnson et al., 2005). Thus, it is necessary and imperative to understand how to improve public employees’ job satisfaction effectively. Previous studies have explored a range of antecedent variables that affect job satisfaction, and scholars in the field of public administration have paid much attention to PSM. PSM is characterized as motives and actions rooted in the public sector to do good for others and the whole society (Perry and Hondeghem, 2008), which consists of four dimensions: attraction to policy making, commitment to the public interest, compassion, and self-sacrifice (Perry, 1996). Research on PSM has been widespread and has reached a consensus on the positive effects of PSM on work attitudes and behaviors (e.g., Liu and Perry, 2016; Ritz et al., 2016; Chen et al., 2021). Many studies have shown that employees with high PSM tend to be more satisfied with their jobs (Naff and Crum, 1999; Bright, 2008, 2021; Andersen and Kjeldsen, 2013).

Following previous research (Bright, 2007, 2021; Wright and Pandey, 2008, 2011), the P-O fit theory is widely used to explain the relationship between PSM and job satisfaction. P-O fit theory assumes that when the congruence between individuals and their organizations increases, employees will have more positive work attitudes and behaviors, such as job satisfaction (Kristof, 1996). In contrast to colleagues with lower PSM, public employees with higher PSM have more tolerance of bureaucratic characteristics, have more excellent compatibility with public organizations, and perceive that the work environment can meet their values, needs, and interests (Scott and Pandey, 2005). It has been proven that PSM can predict P-O fit significantly, leading to higher job satisfaction (Bright, 2007; Teo et al., 2016; Prysmakova and Vandenabeele, 2020). Previous studies have also demonstrated that employees with high PSM will identify more with the public sector (Liu and Perry, 2016) and have a high organizational commitment (Park, 2020), which leads to more job satisfaction. Otherwise, the motivational theory posits that PSM can help public employees find more job enjoyment and express greater satisfaction in daily work (Liu et al., 2008). All these arguments stand to reason that PSM is positively linked to job satisfaction.

Despite the aforesaid positive relationship between PSM and job satisfaction, there are still some inconsistencies in the literature. Based on a survey of 455 council workers in Australia, Rayner et al. (2018) reported a non-significant relationship between PSM and job satisfaction. Considering the “too-much-of-good-thing” effect, several studies proposed that PSM might negatively influence job satisfaction. For example, in people-changing organizations, employees with strong social impact potential would be willing to do too much and overreach individual resources, leading to less satisfaction (van Loon et al., 2015). Meanwhile, the relationship between PSM and tenure also provides supporting evidence. If PSM is positively associated with job satisfaction, then highly satisfied employees are more likely to work longer in public organizations, which conflicts with the negative empirical results between tenure and job satisfaction (Naff and Crum, 1999; Moynihan and Pandey, 2007; Liu and Perry, 2016). Consistent with the rationale of the P-O fit theory, we believe that PSM predicts job satisfaction positively rather than vice versa. Thus, we propose the following hypothesis:

*Hypothesis 1*: PSM has a positive effect on job satisfaction.

### 2.2. The mediating effect of role overload

Role stress refers to the disparity between perceived role expectations and the actual accomplishments in the role (Lambert and Lambert, 2001), which comprises three facets: role conflict, role ambiguity, and role overload (Abdel-Halim, 1981). Role overload has received less attention than role conflict and ambiguity, which have been well-studied (Örtqvist and Wincent, 2006). Role overload depicts situations where employees lack time and resources to complete assigned requirements, commitments, or obligations (Peterson et al., 1995). When it occurs to the relationship between PSM and role overload, the Job Demands-Resources model (JD-R model, Bakker and Demerouti, 2007) can shed some light. PSM can be viewed as job resource which can help public employees cope with tremendous job demands (Giauque et al., 2013). As PSM increases, employees have more personal resources, feel the balance between job demands and resources, and thus are less likely to feel role overload. Otherwise, it has been proven that PSM is positively linked to person-organization fit (Bright, 2007, 2021; Kim, 2012). That is to say, public employees with high PSM tend to feel a high person-organization fit, perceive organizational support, and are inclined to experience less risk of role overload.

PSM can make employees feel more satisfied with their job by decreasing their perception of role overload. From the perspective of the JD-R model, employees who experience role overload will consume their psychological and physiological resources, resulting in decreased well-being, such as job satisfaction. The relationship between role overload and job satisfaction has been widely discussed and reported negative correlations (e.g., Fox et al., 1993; Yousef, 2002; Jones et al., 2007). For instance, based on a survey of 442 public employees, Pecino et al. (2019) reported a significant and negative relationship between role overload and job satisfaction. Meta-analysis integrating nine studies with 2,199 respondents also demonstrated a significant negative relationship between role overload and job satisfaction (Örtqvist and Wincent, 2006). The work–family literature also sheds some light on the relationship between role overload and job satisfaction. Role overload is modeled as an important antecedent of work–family conflict (Duxbury et al., 2021). And work–family conflict can predict low job satisfaction (Bruck et al., 2002). With the increasing nonwork demands like having a toddler and caring for an elder, employees are more likely to experience role overload and perceive work–family conflict, which in turn damages work attitudes and behaviors (Duxbury et al., 2008). In addition, role overload has a negative relationship with work-family enrichment, which can also lead to low job satisfaction (Lapierre et al., 2018).

As stated above, we propose a negative relationship between PSM and role overload and a negative relationship between role overload and job satisfaction. The previous study has pointed out that the impact of personal or organizational resources on employees’ attitudes and behaviors was usually mediated by role overload (Gurbuz et al., 2013; Zhou et al., 2020). Thus, we posit that role overload is an important mediator in the relationship between PSM and job satisfaction. PSM can help employees balance out job demands and resources (Bright, 2007, 2021; Kim, 2012; Giauque et al., 2013), then employees will perceive less role overload and, in turn, enhance the level of job satisfaction. Therefore, we propose the following hypothesis:

*Hypothesis 2*: Role overload mediates the relationship between PSM and job satisfaction.

### 2.3. The moderating effect of marital status

Every individual in society plays multiple roles simultaneously, with work and family roles being the most common roles (Grandy et al., 2005). Personal resources are assumed to be finite. If resources used in one role are depleted, they cannot be used in other roles (Edwards and Rothbard, 2000). Marital status, as a valued life condition in family role (Hobfoll, 1989), has a double-edged nature. On the one hand, in contrast to unmarried employees, married employees usually play more roles. They need to consume more resources to cope with increasing family demands, which may sap time and energy that could be used in the work domain and experience a lower level of job satisfaction. Extant research proposed that due to the shift of resources from work to family, marital status was positively related to work–family conflict (Byron, 2005; Vatharkar and Aggarwal-Gupta, 2020), which may negatively predict job satisfaction (Bruck et al., 2002; Grandy et al., 2005).

On the other hand, when employees are experiencing negative impacts because of a loss of resources in one domain, resources from other domains can mitigate the negative effects (Taris et al., 2001). Marriage can also be regarded as an important form of resources in family context (Hobfoll, 1989), and can shift valued resources to one’s work life (Ten Brummelhuis and Bakker, 2012). Faced with a high level of role overload, married employees could communicate with their spouses to get emotional or verbal appreciation, or perceive helpful advice, thus relieving work tension (Greenhaus et al., 2012). With the increasing commonness of dual-career families (Elloy and Smith, 2004), husband and wife usually share housework. When one of them is busy with work, the other typically takes up more chores, which gives employees a high work-life balance and helps them better deal with challenges at work. Meanwhile, marriage may impose more responsibilities and make employees prefer stable jobs. And they tend to take measures to cope with or adjust unsatisfactory aspects of their work (Azim et al., 2013). It has been proven that married employees enjoyed a higher level of job satisfaction than their single coworkers (Austrom et al., 1988; Azim et al., 2013; Olatunji and Mokuolu, 2014). Previous studies have demonstrated that marital status moderated the effect of role overload on its outcomes by providing additional resources. For example, marital status played moderating role in the relationship between role overload and work engagement (Kim et al., 2018), as well as the relationship between role overload and the work-family interface (Vatharkar and Aggarwal-Gupta, 2020).

Thus, we posit that marital status plays a buffering role in the relationship between role overload and job satisfaction. Specifically, in low role overload, married employees may face additional demands from family life and be less satisfied with their jobs than unmarried employees. In high role overload, married employees may derive emotional support from spouses, do less housework, take the initiative to adjust or adapt unsatisfactory facets to get a steady job, and thus be more satisfied with their jobs than unmarried employees. These lead us to:

*Hypothesis 3*: Marital status moderates the relationship between role overload and job satisfaction in that marital status attenuates the negative effect of increasing role overload.

The above three hypotheses imply an indirect effect of PSM on job satisfaction through role overload, and marital status moderates the relationship between role overload and job satisfaction. Synthesizing the above arguments and hypotheses, we propose a moderated mediation model in which the indirect relationship between PSM and job satisfaction *via* role overload depends on marital status. Thus, we hypothesize that:

*Hypothesis 4*: Marital status moderates the indirect effect of PSM on job satisfaction through role overload; specifically, the relationship is weaker for married public employees than unmarried public employees.

## 3. Methods

### 3.1. Sample

This study collected data from public employees in a medium city in eastern China. With the help of the administration department, we distributed anonymous questionnaires to public employees from various departments, including education, personnel, health, and so on. And they were also informed it is voluntary to join the survey. A total of 500 questionnaires were distributed, and 349 valid questionnaires were obtained after excluding missing data, with a response rate of 69.8%. Among 349 respondents, 40.97% were male, and 59.03% were female. The average age was 35.33. In terms of education level, 74.5% got a bachelor’s degree, and 10.32% got a master’s degree and above. For marital status, 23.78% were unmarried, and 76.22% were married.

### 3.2. Measures

All key variables were measured using Likert five-point scales, ranging from 1 to 5, indicating “strongly disagree” to “strongly agree.”

#### 3.2.1. Public service motivation

This variable was measured by an 18-item scale developed by Liu and Perry (2016), which was validated in the Chinese public sector context. A sample item was “Meaningful public service is very important to me.” The Cronbach’s α for public service motivation was 0.97.

#### 3.2.2. Role overload

Five-item scale developed by Peterson et al. (1995) was used to capture role overload. A representative item was “My workload is heavy.” The Cronbach’s α for role overload was 0.93.

#### 3.2.3. Job satisfaction

This variable was measured by a three-item scale developed by Bono and Judge (2003), which was revised and validated by Liu et al. (2008) in the Chinese context. A sample item was “I feel fairly satisfied with my present job.” The Cronbach’s α for job satisfaction is 0.96.

#### 3.2.4. Marital status

It was measured with one item that asked respondents about their marital status (unmarried = 1, married = 2).

Following previous research (e.g., Liu and Perry, 2016; Sun, 2021), this study controlled demographic variables, including gender (male = 1, female = 2), age, education (college and below = 1, bachelor =2, master and above = 3), and organizational tenure (in years).

## 4. Results

### 4.1. Preliminary analysis

Before hypothesis testing, this study did some preliminary analysis. Firstly, since our data was self-reported data collected at one time, Harman’s single-factor test was conducted to examine common method bias (CMB). Four factors were drawn, and the first factor explained 30.75% of the total variance, which did not account for most of the variance, implying that CMB wasn’t a substantial concern (Podsakoff et al., 2003).

Table 1 presents the result of the confirmatory factor analysis. The results demonstrate that the three-factor model (public service motivation, role overload, and job satisfaction) fits well with the data (*χ*^2^ = 187.48, df = 51, *χ*^2^/df = 3.68, CFI = 0.97, TLI = 0.96, RMSEA = 0.09, SRMR = 0.03). It performs better than the two-factor and one-factor models on all fitting indicators, indicating that the three constructs have good structural validity.

**Table 1:** Confirmatory factor analysis (CFA) results.

Model	*χ* ^2^	*df*	CFI	TLI	RMSEA	SRMR
Three-factor model (PSM, RO, and JS)	187.48	51	0.97	0.96	0.09	0.03
Two-factor model (PSM and JS combined, RO)	1122.01	53	0.74	0.68	0.24	0.12
One-factor model (PSM, RO, and JS combined)	2466.18	54	0.42	0.29	0.36	0.25

PSM, public service motivation; RO, role overload; JS, job satisfaction.

### 4.2. Descriptive statistics and correlation analysis

Table 2 reports the research variables’ means, standard deviations, and correlation coefficients. According to the results of Table 2, public service motivation is positively correlated with job satisfaction (*r* = 0.57, *p* < 0.001) and negatively correlated with role overload (*r* = −0.16, *p* < 0.01). Table 2 also shows that the relationship between role overload and job satisfaction is significantly negative (*r* = −0.31, *p* < 0.001). Thus, correlations between main variables provide initial support for research hypotheses.

**Table 2:** Means, standard deviations, and correlations.

Variable	Mean	*SD*	1	2	3	4	5	6	7
1. Gender	1.59	0.49							
2. Age	35.33	8.06	−0.19^***^						
3. Education	1.95	0.50	−0.09	−0.21^***^					
4. Tenure	7.69	7.74	−0.02	0.69^***^	−0.16^**^				
5. PSM	4.39	0.51	−0.02	−0.09	0.14^**^	−0.12^*^			
6. RO	3.21	0.88	−0.12^*^	0.07	0.01	0.14^*^	−0.16^**^		
7. Marital status	1.76	0.43	−0.12^*^	0.53^***^	−0.07	0.31^***^	−0.01	−0.01	
8. JS	4.10	0.73	−0.08	0.01	0.07	−0.04	0.57^***^	−0.31^***^	0.03

*N* = 349, *p < 0.05, **p < 0.01, ***p < 0.001. Gender is coded as male = 1, female = 2. Marital status is coded as unmarried = 1, married = 2. PSM, public service motivation, RO, role overload, JS, job satisfaction.

### 4.3. Hypothesis testing

Table 3 shows the results of the regression analysis. Model 1 has controlled demographic variables to test the relationship between PSM and job satisfaction. The results show that PSM positively affects job satisfaction (*β* = 0.57, *p* < 0.001) and explains 33% of the variance. Thus, Hypothesis 1 is supported.

**Table 3:** Results of regression analysis.

	JS	RO	JS	JS	JS
	M1	M2	M3	M4	M5
Gender	−0.06	−0.13^*^	−0.09^*^	−0.10	−0.08
Age	0.05	−0.09	0.03	0.03	0.04
Education	−0.01	0.03	−0.00	0.06	−0.01
Tenure	−0.01	0.18^*^	0.03	−0.02	0.02
PSM	0.57^***^	−0.15^**^	0.54^***^		0.54^***^
RO			−0.24^***^	−0.33^***^	−0.25^***^
Marital status				0.01	−0.01
RO*Marital status				0.12^*^	0.14^**^
*R* ^2^	0.33	0.06	0.39	0.13	0.41

*N* = 349, *p < 0.05, **p < 0.01, ***p < 0.001. Gender was coded as male = 1, female = 2. Marital status was coded as unmarried = 1, married = 2. PSM, public service motivation, RO, role overload, JS, job satisfaction.

Then, following Baron and Kenny’s (1986) recommended procedures, Models 2 and 3 are employed to test the mediation effect. As model 2 shows, PSM is negatively associated with role overload (β = −0.15, *p* < 0.001). Model 3 adds role overload based on Model 1. The results demonstrate a significantly negative effect of role overload on job satisfaction (β = −0.24, *p* < 0.001), and PSM is still positively associated with job satisfaction (β = 0.54, *p* < 0.001). The inclusion role overload explains 6% of the variance for job satisfaction (from Model 1 (*R*^2^ = 0.33) to Model 3 (R^2^ = 0.39)). Hypothesis 2 is supported.

**Figure 1:**
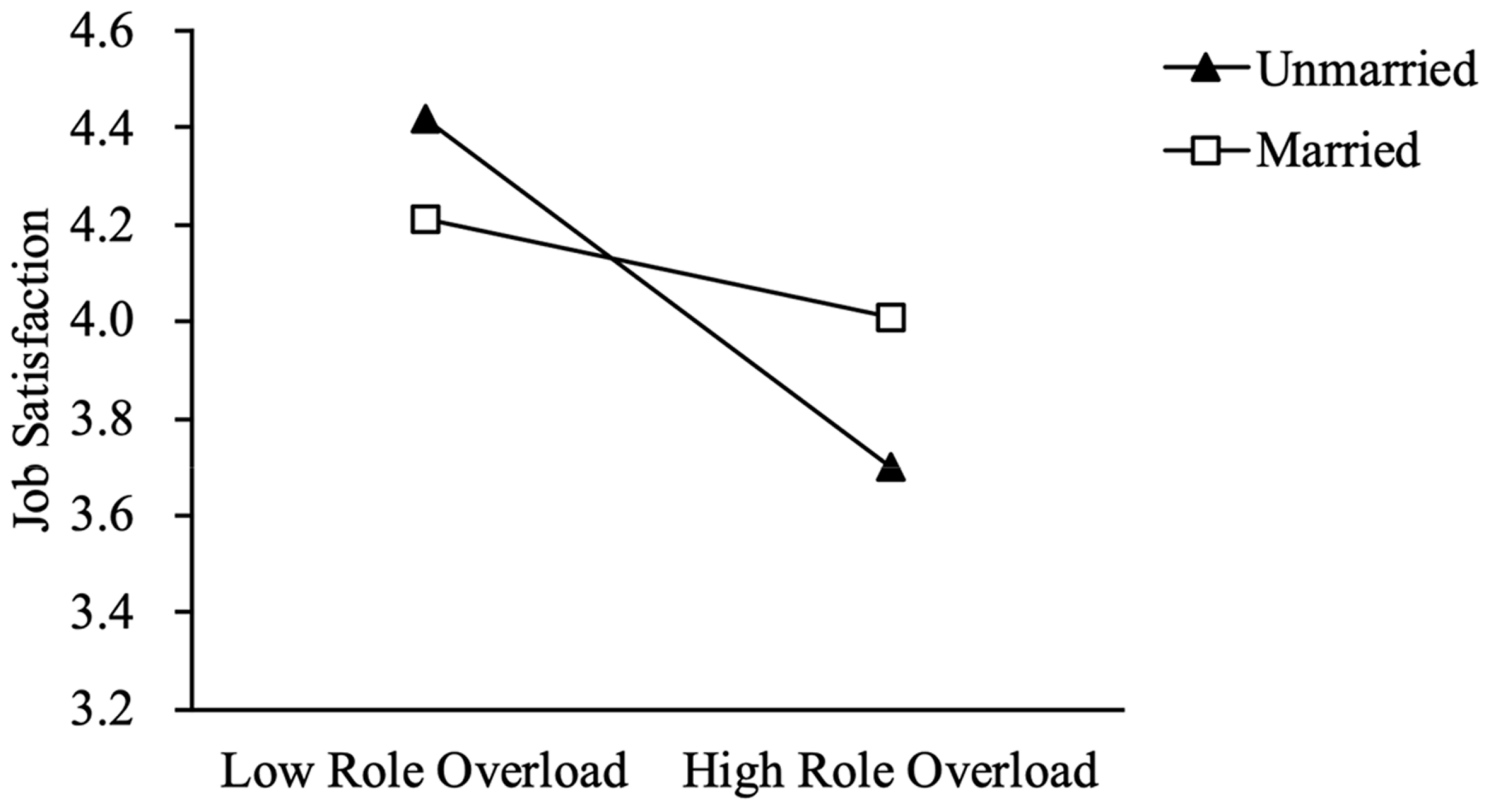
The moderating effect of marital status on the relationship between role overload and job satisfaction.

Model 4 includes the interaction term between role overload and marital status to test the moderation effect of marital status on the relationship between role overload and job satisfaction. The results report that the interaction term is positively associated with job satisfaction (β = 0.12, *p* < 0.05) and explains 13% of the variance. Hypothesis 3 is supported. Model 5 includes the interaction term to Model 3 to test Hypothesis 4. The results show that the interaction term is positively associated with job satisfaction (β = 0.14, *p* < 0.01), which explains 2% of the variance for job satisfaction (Model 3 (*R*^2^ = 0.39) to Model 5 (*R*^2^ = 0.41)). Hypothesis 4 is supported. Figure 1 displays the moderation effect of marital status on the relationship between role overload and job satisfaction. As Figure 1 shows, the slope of unmarried employees is negative and steeper, implying that the relationship between role overload and job satisfaction is negative and intense. In contrast, the slope of married employees is negative and flatter. Specifically, when employees are faced with a higher level of role overload, married employees will experience a higher level of job satisfaction than unmarried employees.

To further examine the Hypothesis more rigorously, we perform bootstrapping analysis with PROCESS macro for SPSS. Table 4 shows the results of the mediation effect and the moderated mediation effect. Model 4 in PROCESS is used to test the mediation effect, and Model 14 in PROCESS is used to examine the moderated mediation effect. As Table 4 shows, the indirect impact of PSM on job satisfaction through role overload is significant, with 95% CI [0.02, 0.09]. And 95% direct CI is [0.64, 0.88], excluding zero. Thus, role overload partially mediates the relationship between PSM and job satisfaction. Hypothesis 2 is supported.

**Table 4:** Results of bootstrap analysis.

Outcome		Effect	Boot SE	95% CI	
				Lower	Upper
Direct effect		0.76	0.06	0.64	0.88
Indirect effect		0.05	0.02	0.02	0.09
Conditional indirect effect	Unmarried	0.11	0.04	0.03	0.20
	Married	0.03	0.01	0.01	0.06
	Group difference	−0.08	0.04	−0.16	−0.02

Bootstrap = 5,000; *N* = 349; CI = confidence interval.

The results of the conditional indirect effect of marital status are also displayed. For unmarried public employees, the indirect effect of PSM on job satisfaction does not include zero, 95% CI is [0.03, 0.20], implying a significant indirect effect. For married public employees, the indirect effect of PSM on job satisfaction also does not include zero, 95% CI is [0.01, 0.06], implying the indirect effect is significant. And the moderated mediation effect (difference between two groups) is significant, with 95% CI [−0.16, −0.02]. Therefore, Hypothesis 4 is supported.

**Figure 2:**
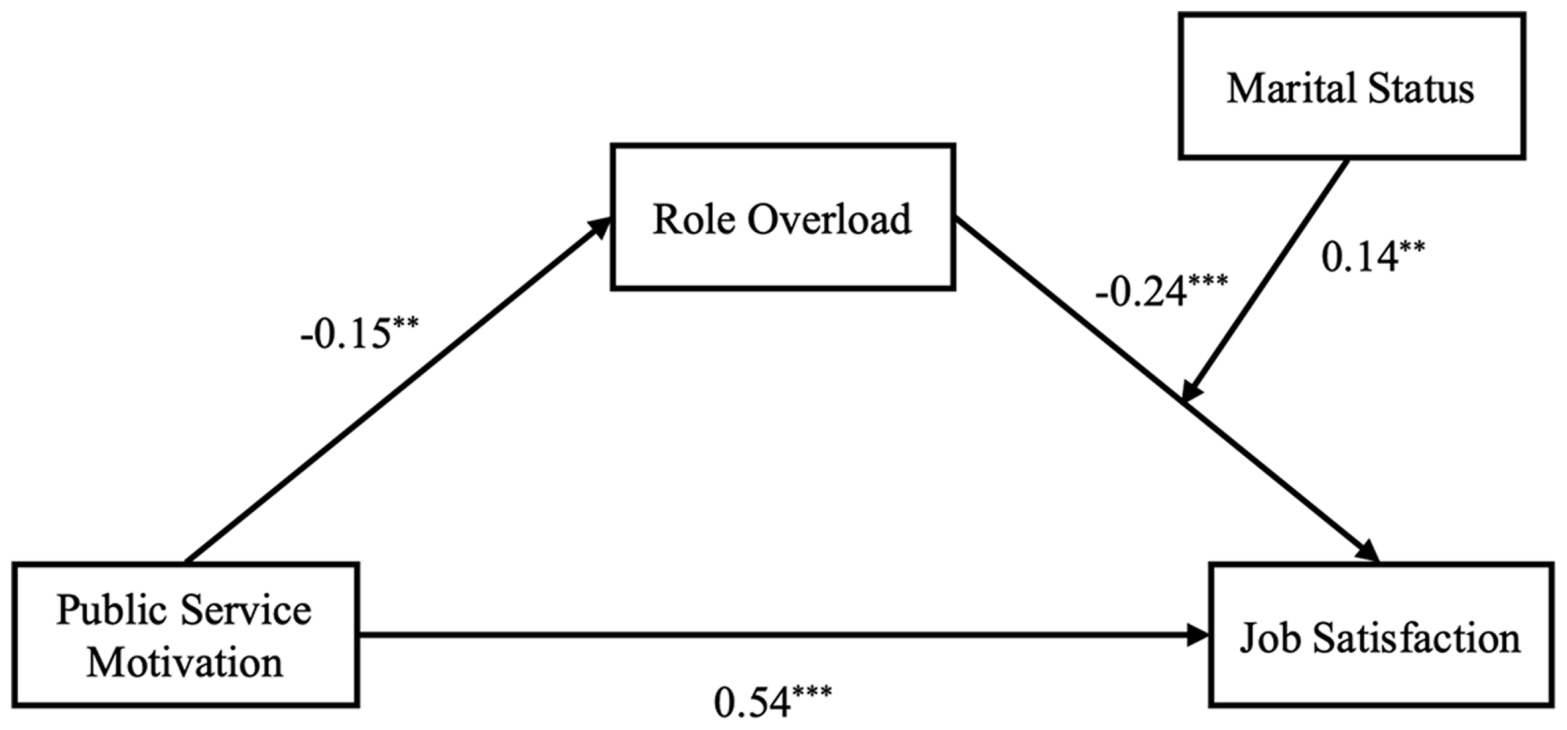
Result of theoretical model. *p<0.05, **p<0.01, ***p<0.001.

Figure 2 presents the results of the proposed moderated mediation model.

## 5. Discussion

With increasing efficiency and high-quality requirements for the public sector, how to improve public employees’ job satisfaction has been a critical theoretical and practical issue (Cantarelli et al., 2016). Although scholars have extensively examined the relationship between PSM and job satisfaction, little attention has been paid to its psychological mechanism and boundary condition. This study confirms the positive relationship between PSM and job satisfaction and examines the mediating effect of role overload and the moderating effect of marital status. The findings of this study provide both theoretical and practical implications.

### 5.1. Theoretical implication

Firstly, this study advances the development of public service motivation theory. Previous studies have widely examined the relationship between PSM and job satisfaction, leading to contradictory results (e.g., Naff and Crum, 1999; Van Loon et al., 2015; Rayner et al., 2018). In line with previous findings (Liu et al., 2008; Liu and Tang, 2011), we confirm a positive direct relationship between PSM and job satisfaction based on an investigation among 349 Chinese public employees. Meanwhile, compared to vast attention on the relationship between PSM and two kinds of role stress, role ambiguity and role conflict, little attention has been paid to the relationship between PSM and role overload. Only Gould-Williams et al. (2014) took civic duty as a primary component of PSM and investigated the relationship between work overload and civic duty. To the best of our knowledge, our study is the first empirical research to examine the relationship between PSM and role overload. It demonstrates that employees with high PSM are inclined to perceive role overload. Some studies conflict with our findings. Scholars propose that high PSM employees might fully engage in their work, perform extra-role behavior (Bolino and Turnley, 2005; Giauque et al., 2013), which requires additional time and energy, and thus are more prone to role overload. Lin and Ling (2018) thought that role overload belonged to challenge stress, which is positively associated with PSM (Deng et al., 2019, 2021). Thus, future research should further explore the relationship between PSM and role overload, such as the curvilinear relationship.

Secondly, this study has enriched the mediating variables between PSM and job satisfaction. Although previous studies have examined some potential mediators in the relationship between PSM and job satisfaction (Wright and Pandey, 2008, 2011; Li and Wang, 2016; Park, 2020; Bright, 2021), rare studies were carried out from the perspective of role overload. Noteworthy, the results of our research show that role overload only partially mediated the relationship between PSM and job satisfaction. Future research, therefore, should explore more mediators. Role overload can be split into two types: quantitative role overload and qualitative role overload (Yousef, 2002). The former refers to time limitation; the latter emphasizes a lack of ability, skill, and knowledge. Two kinds of role overload should be taken into account in future research. In addition, this study only focuses on the impact of role overload on the work domain. Employees who perceive role overload may bring work home and occupy personal time they could spend with family, which certainly influences the family domain (Carnes, 2017). Previous studies claimed dynamic spillover between work satisfaction and marriage satisfaction (Heller and Watson, 2005). Thus, future research could examine the impact of role overload on the family domain.

Thirdly, this study depicts the boundary condition and incorporates the work and family domains by examining the moderating effect of marital status. Although married employees can drain resources from their family domain, there is still a negative relationship between role overload and job satisfaction, implying that the amount of resources marriage provides is limited (Lapierre et al., 2018). In addition, the demands of the family domain are complex and changeable in the Chinese context. According to the China Health and Retirement Longitudinal Study (CHARLS) 2011–2012, about one-third of Chinese families are multi-generational co-residence which implies that Chinese families are faced with more demands like caring for the elder (Xie, 2013). Having a toddler can also limit the resources employees can get from their families. Families with children under 6 years old have been found to report more family–work conflict (Bennett et al., 2017). Moreover, males and females are considered to play different roles in traditional Chinese culture. For instance, the husband is usually perceived as a breadwinner, and the wife is thought to deal with housework. Some scholars suggested that the wife may experience higher work–family conflict than the husband do (Wharton and Blair-Loy, 2006). Interestingly, the increase in education has eroded traditional gender values (Hu and Scott, 2016). Therefore, future research should control the influence of the national context.

### 5.2. Practical implication

First of all, this study confirmed that public employees with high PSM were more satisfied with their jobs, which raised the importance of cultivating PSM in the public sector. As a low-cost driver of job satisfaction (Homberg et al., 2015), PSM could be cultivated in various ways. For example, the public organization can implement standard tests to select individuals with high PSM and facilitate workplace trust to enhance public employees’ PSM (Paarlberg et al., 2008; Chen et al., 2021). Public organizations should also provide opportunities to serve the public, which was considered to enhance the positive effect of PSM on job satisfaction (Homberg et al., 2015). However, it is worth noting that PSM is not a panacea for high job satisfaction (Bright, 2008). Previous studies have proved that PSM might have a dark side, lead to “resigned satisfaction,” work stress, unethical behavior, and so on (Giauque et al., 2012, 2013; Liu et al., 2015a,b; Ripoll and Schott, 2020).

The findings of this study show that role overload partially mediated the relationship between PSM and job satisfaction, suggesting that public employees’ job satisfaction can be increased by eliminating role overload. Extant research has provided many management insights to decrease role overload. It is proved that leader-member exchange quality can offer additional resources to public employees and negatively predicts role overload (Tordera et al., 2008). Thus, Public organizations should take measures to cultivate high-quality relationships between leaders and employees. And total quality management (TQM) practice, like encouraging employees to involve in the decision-making process, can mitigate role overload (Ebrahimi et al., 2014). It has been proven that job involvement can buffer the negative effect of role overload (Allen et al., 2001).

Thirdly, our results show that marital status moderates the relationship between role overload and job satisfaction and the indirect relationship between PSM and job satisfaction. When employees are faced with high role overload, marriage can shift time and energy from the family domain to the work domain, help employees cope with tremendous demands, and increase their job satisfaction. But in the long run, the marriage hardly supplies resources sustainably, and growing marital discord may reduce job satisfaction (Rogers and May, 2003). Thus, it is vital to achieve a work-family balance. Thus, achieving work-family balance is vital, especially in enhancing positive spillover. Employees could gain resources like multiple-task skills, networking, and positive emotions from family lives, leading to higher job satisfaction (Greenhaus and Powell, 2006). Employees should cultivate a good family environment, like family cohesion and mutuality, which positively relates to positive family-to-work spillover (Carlson et al., 2006; Stevens et al., 2007). Meanwhile, public organizations should have formal institutional support to help employees balance work and family roles, such as flexible working arrangements, teleworking, time management training programs, and childcare facilities (McCarthy et al., 2010). Also, the public sector should foster family-supportive supervisor behaviors, which are essential social resources that can assist employees in managing demands from family and work and enhance employees’ well-being (Kossek et al., 2011).

### 5.3. Limitations and future research

Future research should address several limitations. First, data were collected from a convenience sample in a medium city in eastern China. It has been suggested that the relationship between PSM and its correlations may be influenced by cultural context (Houston, 2011; Rayner et al., 2018). And previous research also showed that the effect of role overload on organization commitment was contingent on power distance (Fisher, 2014). Future research should be conducted in larger samples to increase the generalizability of findings. The second limitation of this study is the cross-sectional design, which makes it challenging to clarify the causal relationship. Longitudinal or experimental research design is expected in future research. As well, all variables were self-reported. Future research could use different data sources, such as objective data, to decrease the risk of common method bias. Third, this study ignored the complexity of family domain demands and resources, such as the number of children, the need for elder care, and the complexity of marital status like unmarried cohabitation. Future research needs to consider different family compositions.

## 6. Conclusion

This study focused on the relationship between PSM and job satisfaction to investigate how to enhance public employees’ well-being effectively. Based on a survey of 349 public employees in eastern China, results showed that PSM had a significant positive effect on public employees’ job satisfaction and role overload plays a mediation role in their relationship. These findings not only respond to previous contradictory results and contribute to the development of role stress theory, but also advance the development of public service motivation theory by clarifying the psychological mechanism of PSM with job satisfaction and bringing insight into how PSM influences employees’ job satisfaction. Results also revealed that marital status played a moderation role in the relationship between role overload and job satisfaction and the indirect relationship between PSM and job satisfaction. Through depicting the boundary condition, marital status deepens our understanding of resources spillover between work and family domains. Under the imperative circumstance of increasing employees’ well-being, public organizations should select and foster PSM of employees, develop high Leader-member exchange quality and total quality management to eliminate role overload, and cultivate formal institutional support and family-supportive supervisor behaviors.

## Data availability statement

The original contributions presented in the study are included in the article/supplementary material, further inquiries can be directed to the corresponding author.

## Author contributions

The author confirms being the sole contributor of this work and has approved it for publication.

## Funding

This work was supported by the National Social Science Fund of China (Project 20AZD020).

## Conflict of interest

The author declares that the research was conducted in the absence of any commercial or financial relationships that could be construed as a potential conflict of interest.

## Publisher’s note

All claims expressed in this article are solely those of the authors and do not necessarily represent those of their affiliated organizations, or those of the publisher, the editors and the reviewers. Any product that may be evaluated in this article, or claim that may be made by its manufacturer, is not guaranteed or endorsed by the publisher.
